# Association Between Folic Acid Supplementation and Retinal Atherosclerosis in Chinese Adults With Hypertension Complicated by Diabetes Mellitus

**DOI:** 10.3389/fphar.2018.01159

**Published:** 2018-10-30

**Authors:** Ying Meng, Jun Li, Xuling Chen, Haicheng She, Liang Zhao, Yuan Peng, Jing Zhang, Kun Shang, Haibo Li, Wenbin Yang, Yadi Zhang, Xiaopeng Gu, Jianping Li, Xianhui Qin, Binyan Wang, Xiping Xu, Fanfan Hou, Genfu Tang, Rongfeng Liao, Liu Yang, Yong Huo

**Affiliations:** ^1^Key Laboratory of Vision Loss and Restoration, Ministry of Education, Ophthalmology, Peking University First Hospital, Beijing, China; ^2^Peking University International Hospital, Beijing, China; ^3^Peking University First Hospital, Beijing, China; ^4^Beijing Ophthalmology and Visual Science Key Laboratory, Beijing Tongren Eye Center, Beijing Tongren Hospital, Capital Medical University, Beijing, China; ^5^Institute of Biomedicine, Anhui Medical University, Hefei, China; ^6^Nanfang Hospital, Southern Medical University, Guangzhou, China; ^7^First Affiliated Hospital of Anhui Medical University, Hefei, China

**Keywords:** retinal atherosclerosis, folic acid, hypertension, diabetes mellitus, homocysteine

## Abstract

**Background**: This cross-section investigation included 2,199 participants with hypertension complicated by diabetes mellitus, a cohort of the China Stroke Primary Prevention Trial in which 20,702 patients with essential hypertension were given enalapril with folic acid or enalapril-only double-blind treatment for 5 years. This study aimed to explore the correlation between folic acid supplementation and retinal atherosclerosis (RA) in adults with hypertension complicated by diabetes mellitus.

**Methods:** The diagnosis of RA was determined by non-mydriatic fundus photography and classified by the Keith-Wagener-Barker system. The statistical correlation of folic acid supplementation with RA prevalence and severity was assessed.

**Results:** Of our cohort, 1,698 (77.6%) participants were diagnosed with RA, and the prevalence in males and females was 78.0 and 75.6%, respectively. Participants in the enalapril group had higher total homocysteine (tHcy) levels than those in enalapril–folic acid group. Compared with the enalapril group in the tHcy > 15 μmol/L group of females, the odds ratio for the enalapril–folic acid group was 0.28 (95% confidence interval, 0.11–0.67, *P* = 0.0061).

**Conclusions:** The prevalence of RA was high (77.6%) in our cohort of adults with hypertension complicated by diabetes mellitus. Folic acid supplementation was significantly associated with reduced risk of RA in females with hyperhomocysteinemia. No significant association were seen in males.

## Introduction

Hypertension and diabetes cause noninfectious and retrograde pathological changes, or atherosclerosis, in the retinal arteries. Those changes are closely related to pathological changes in the vessels of other organs. The retinal blood vessels are the only directly observable vascular system in the human body, making them valuable for evaluating damage in target organs such as heart, kidney, and brain (Cheung et al., 2012; Ong et al., 2013; Kabedi et al., 2014).

Blood pressure (BP) and plasma glucose levels are important risk factors of retinal atherosclerosis (RA) (Hubbard et al., 1999; Wong et al., 2004). There is a positive correlation between BP, plasma glucose, and the incidence of atherosclerosis. Study shows that homocysteine (Hcy) level is also a critical risk factor of RA (Ghorbanihaghjo et al., 2008). A common feature of hypertension patients, especially those in China, is that they have higher Hcy levels (Hao et al., 2007; Chen et al., 2017). Many studies show that hypertension and high Hcy levels have synergistic effect in causing cardiovascular and cerebrovascular events (Bortolotto et al., 1999; Sharabi et al., 1999; Han et al., 2015). Reducing BP or Hcy level alone may not be sufficient to reduce the risk of cardiovascular and cerebrovascular events.

Folic acid consumption is the most efficient way to lower Hcy level (1998). The results of a meta-analysis (Wang et al., 2007) showed that 36-month consumption of folic acid and a 20% reduce in Hcy have a notable effect in preventing stokes. The study of the China Stroke Primary Prevention Trial (CSPPT) showed that in Chinese hypertensive adults, an fasting blood glucose concentration ≥7.0 mmol/L or diabetes is associated with an increased risk of first stroke, this increased risk is reduced by 34% with folic acid treatment (Xu et al., 2017). A large scale randomized, double blind and placebo controlled Women's Antioxidant and Folic Acid Cardiovascular Study (WAFACS) (Albert et al., 2008) confirms that angiotensin converting enzyme inhibitors (ACEI) and folic acid have a synergistic effect in reducing the risks of cardiovascular and cerebrovascular events. The effect is not shown in other antihypertensive drugs.

Most of the studies nowadays on the reduction of Hcy with folic acid focus on the effects on cardiovascular and cerebrovascular diseases, but not on the effects on RA, particularly those of patients with hypertension and diabetes. Our study focuses on the correlation of folic acid consumption of patients with hypertension and diabetes and their incidence of RA.

## Materials and methods

### Study settings and participants

All subjects in this study came from the exit visit of the China Stroke Primary Prevention Trial (CSPPT; clinical trials.gov identifier: NCT00794885), a large community-based, randomized, multicenter, double-blind, and actively controlled trial with a total of 20,702 participants. The study complied with the Helsinki Declaration and was approved by the Ethics Committee of the Institute of Biomedicine, Anhui Medical University, Hefei, China (FWA assurance number FWA00001263). All participants provided written informed consent. The CSPPT was designed to evaluate whether combination therapy with enalapril maleate and folic acid tablets was more effective at preventing stroke in Chinese adults with hypertension than enalapril maleate alone. Participants were randomly assigned to receive double-blind daily treatment with a single-pill combination containing 10 mg of enalapril and 0.8 mg of folic acid or a tablet containing 10 mg of enalapril alone. Participants in the CSPPT study were “relatively healthy” hypertensives without a history of myocardial infarction, stroke, heart failure, cancer, or other serious mental disorders. Details regarding inclusion/exclusion criteria, treatment assignment, and outcome measures of the trial can be viewed at http://clinicaltrials.gov/ct2/show/NCT00794885.

**Figure 1:**
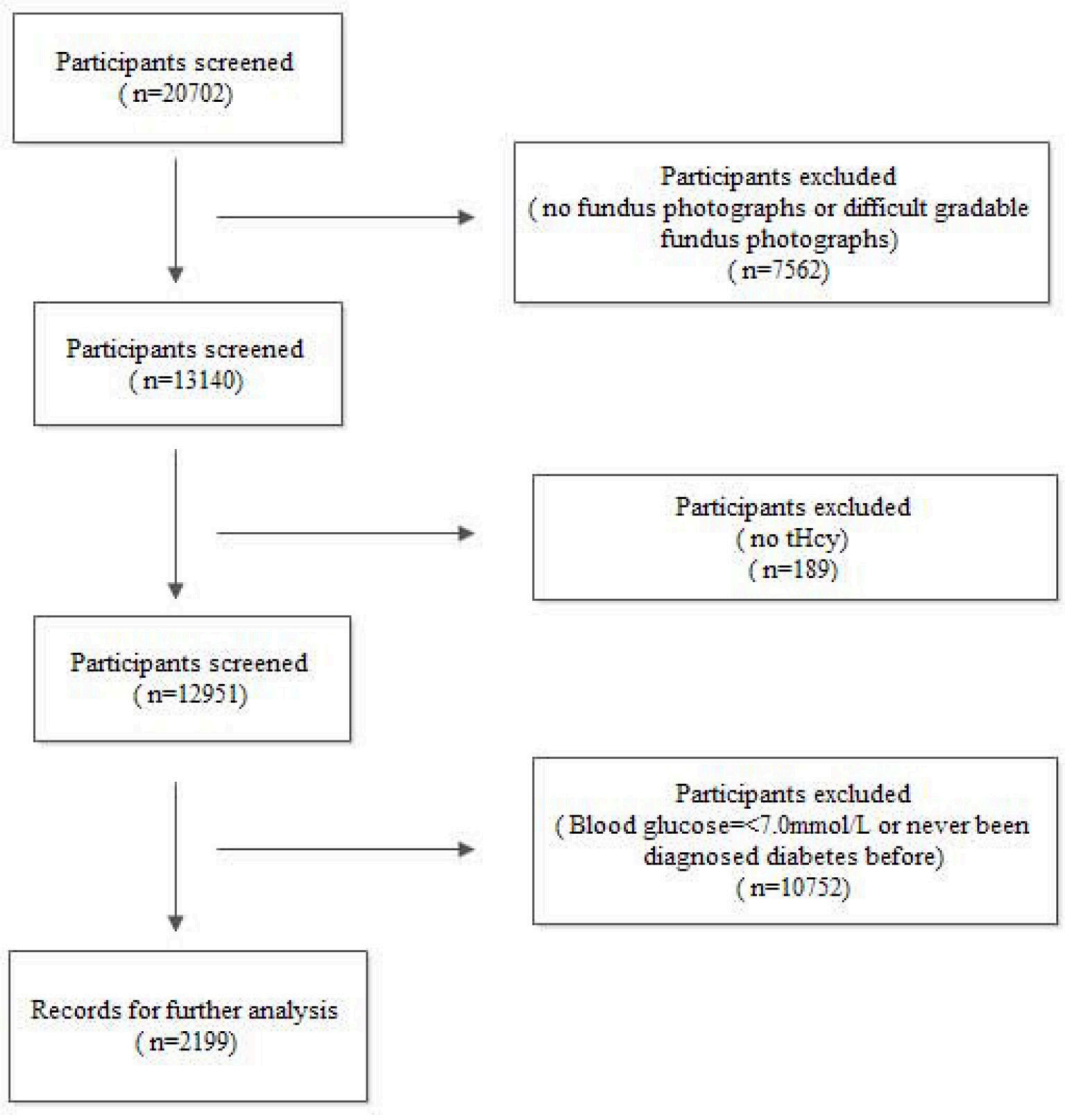
Flow diagram of the screening and enrollment of study participants.

All subjects in this study came from the China Stroke Primary Prevention Trial (CSPPT), conducted from May 19, 2008, to August 24, 2013, in 32 communities in the Jiangsu and Anhui provinces of China. Among the 20,702 participants, 7,562 were excluded because of missing or low-quality fundus photographs; 10,752 were excluded because their fasting plasma glucose level was < 7 mmol/L or they had never been diagnosed with diabetes; and 189 were excluded due to unavailable Hcy levels. Therefore, the remaining 2,199 subjects were analyzed (Figure 1).

### RA classification

Non-mydriatic fundus photographs were taken in the posterior pole and macula-centered using fundus cameras (Topcon TRC-NW8 Non-Mydriatic Retinal Camera, Canon CR-2 AF Non-Mydriatic Retinal Camera, and Kowa Nonmyd 7 Fundus Camera). All photographs were randomly evaluated by four ophthalmologists with double-blinded. Consistency checks (kappa value, 0.71–0.95) prove the reliability of our results. RA was classified into four grades according to the Keith-Wagener-Barker system (Keith et al., 1974) (Table 1).

**Table 1:** The Keith–Wagener–Barker classification system for retinal atherosclerosis.

**Grade**	**Feature**
None	No detectable positive signs
1	Mild or moderate generalized retinal arteriolar narrowing, arteriovenous tortuosity
2	Definite focal narrowing and arteriovenous nipping,crossing compression
3	Copper wire or silver wire artery, signs of grade 2 retinopathy plus retinal hemorrhages, exudates and cotton wool spots
4	Severe grade 3 retinopathy plus papilledema or retinal edema

### BP measurements

BP was measured with each subject in a seated position using a mercury sphygmomanometer with an appropriate cuff size. For each subject, BP was measured three times with a 5-min rest period between measurements. The mean of three measurements was recorded for the statistical analyses. Hypertension was defined as a BP > 140 mmHg systolic and/or 90 mmHg diastolic.

### Laboratory examinations

Laboratory examinations were performed at the core lab of the National Clinical Research Center for Kidney Disease (Nanfang Hospital, Guangzhou, China). After a 12–15 h fast, a venous blood sample was obtained from each subject. Serum or plasma samples were separated within 30 min of collection and stored at −70°C. The levels of glucose, Hcy, creatinine, uric acid, blood urea nitrogen (BUN), alkaline phosphatase (ALP), and lipids (including total cholesterol, high-density lipoprotein cholesterol, and triglycerides) of all the collected blood samples were measured.

### Brachial–ankle pulse wave velocity measurements

Brachial–ankle pulse wave velocity (baPWV) was automatically measured using PWV/ABI instruments (BP-203RPE; Omron-Colin, Japan) by trained volunteers recruited from local medical colleges. Occlusion and monitoring cuffs matched with oscillometric sensors were wrapped around the subjects' upper arms and ankles and pulse volume waveforms of the bilateral brachial and tibial arteries were recorded simultaneously to determine the time interval between the initial increase in brachial and tibial waveforms (transit time, ΔTba). The transmission distance from the brachium to the ankle was calculated by body height. The path length from the suprasternal notch to the brachium (Lb) was obtained using the following equation: Lb = 0.2195 × height of the patient (in cm) – 2.0734. The path length from the suprasternal notch to the ankle (La) was obtained using the following equation: La = (0.8129 × patient height [cm] + 12.328). The baPWV value was calculated as the ratio of the transmission distance from the brachium to the ankle divided by the transit time as follows: baPWV = (La – Lb)/ΔTba.

### Demographic data

All participants were interviewed using a standardized questionnaire that assessed for age, sex, sociodemographic status, education, occupation, diet, lifestyle, health behavior, medical history, and personal history including smoking status, alcohol consumption, and known systemic diseases. Anthropometric measurements were taken according to a standard operating procedure. Body mass index (BMI) was calculated as weight (kg) divided by height (m^2^). The waist-to-hip ratio was calculated as the ratio of waist circumference to hip circumference.

### Statistical analysis

All analyses were performed using EmpowerStats (http://www.empowerstats.com) and the statistical package R. Data are presented as mean ± standard deviation or proportion. Stratified analysis, interaction tests, and covariate screenings were performed. Binary logistic regression analyses were used to assess the associations between RA and treatment groups. RA was evaluated as a binary variable; grade 1 to grade 4 participants were merged together for the analysis. Total Hcy (tHcy) levels were evaluated as a continuous variable. The results are shown as odd ratios (OR) and 95% confidence intervals (CI) with adjustment for major variables including age, sex, study center, treatment group, BMI, systolic BP, diastolic BP, triglycerides, fasting plasma glucose, and creatinine. Two-tailed *P* < 0.05 were considered statistically significant.

## Results

The present study included a total of 2,199 participants from the CSPPT (*n* = 1,088 and 1,111 in the enalapril–folic acid and enalapril groups, respectively). Among them, 1,698 (77.6%) had RA, the prevalence of which in males and females was 78.0 and 75.6%, respectively (Supplementary Material). Demographic and anthropometric characteristics and the laboratory results of the participants are listed in Table 2. There were significant differences in mean arterial pressure (MAP; *P* = 0.049) and tHcy (*P* < 0.001) between the two treatment groups by sex and total participants. The enalapril group had higher MAP and tHcy levels than the enalapril–folic acid group. The mean tHcy levels for the enalapril–folic acid and enalapril groups were 12.70 ± 6.69 and 13.93 ± 6.94 μmol/L in total, 14.38 ± 8.72 and 16.19 ± 9.39 μmol/L in males, and 11.74 ± 4.94 and 12.55 ± 4.34 μmol/L in females, respectively.

**Table 2:** Baseline characteristics of the study participants.

	**Total**			**Male**			**Female**		
**Characteristics**	**Enalapril-folic acid *n* = 1,088**	**Enalapril *n* = 1,111**	***P*-value**	**Enalapril-Folic acid *n* = 396**	**Enalapril *n* = 421**	***P*-value**	**Enalapril-folic acid *n* = 692**	**Enalapril *n* = 690**	***P*-value**
Age, years	63.95 ± 7.10	64.18 ± 7.30	0.481	64.42 ± 7.44	64.67 ± 7.52	0.626	63.68 ± 6.88	63.85 ± 7.17	0.657
BMI, kg/m^2^	26.14 ± 3.85	26.17 ± 3.88	0.842	25.28 ± 3.67	25.38 ± 3.84	0.699	26.64 ± 3.86	26.66 ± 3.82	0.910
WHR	0.94 ± 0.06	0.94 ± 0.06	0.613	0.94 ± 0.06	0.94 ± 0.06	0.501	0.93 ± 0.07	0.93 ± 0.06	0.951
SBP, mmHg	136.21 ± 17.22	137.36 ± 17.85	0.123	133.30 ± 16.59	135.64 ± 18.53	0.058	137.88 ± 17.37	138.42 ± 17.34	0.564
DBP, mmHg	80.58 ± 11.17	81.39 ± 11.01	0.098	80.61 ± 11.47	82.29 ± 11.87	0.060	80.56 ± 11.00	80.83 ± 10.42	0.529
MAP, mmHg	99.12 ± 11.44	100.04 ± 11.34	0.049	98.17 ± 11.31	100.07 ± 12.21	0.027	99.67 ± 11.48	100.03 ± 10.78	0.435
tHcy, umol/L	12.70 ± 6.69	13.93 ± 6.94	< 0.001	14.38 ± 8.72	16.19 ± 9.39	0.004	11.74 ± 4.94	12.55 ± 4.34	0.001
Uric acid, umol/L	324.89 ± 96.14	329.70 ± 95.19	0.251	365.22 ± 99.51	363.76 ± 100.98	0.834	301.77 ± 86.07	308.71 ± 85.14	0.132
Creatinine, μmol/L	65.50 ± 21.66	65.68 ± 20.37	0.840	78.41 ± 24.19	77.24 ± 22.43	0.471	58.11 ± 15.92	58.62 ± 15.17	0.543
Triglycerides, mmol/L	2.31 ± 1.77	2.39 ± 2.12	0.354	2.03 ± 1.85	2.18 ± 2.26	0.284	2.47 ± 1.70	2.51 ± 2.01	0.691
HDL-C, mmol/L	1.27 ± 0.33	1.25 ± 0.33	0.187	1.26 ± 0.34	1.24 ± 0.38	0.620	1.27 ± 0.33	1.25 ± 0.29	0.190
Glucose, mmol/L	9.25 ± 3.07	9.40 ± 3.37	0.276	9.24 ± 3.18	9.31 ± 3.13	0.766	9.25 ± 3.01	9.45 ± 3.51	0.252
ALP, U/L	98.40 ± 31.49	97.50 ± 30.76	0.498	86.94 ± 26.25	88.55 ± 26.99	0.391	104.92 ± 32.37	102.93 ± 31.63	0.250
eGFR, ml/min/1.73 m^2^	90.56 ± 15.83	90.46 ± 15.93	0.876	88.47 ± 16.34	89.23 ± 16.99	0.516	91.76 ± 15.41	91.21 ± 15.21	0.503
BUN, mmol/L	6.28 ± 1.98	6.28 ± 1.88	0.962	6.62 ± 2.05	6.62 ± 2.14	0.955	6.09 ± 1.92	6.06 ± 1.66	0.797
PWV, mm/s	1763.02 ± 424.80	1785.90 ± 382.79	0.201	1728.01 ± 424.46	1748.74 ± 406.98	0.501	1782.89 ± 424.04	1808.10 ± 365.67	0.261
Retinal arteriosclerosis, *n* (%)	845 (77.67%)	852 (76.76%)	0.656	311 (78.54%)	343 (81.47%)	0.336	534 (77.17%)	510 (73.91%)	0.179
Cooking oil, *n* (%)			0.801			0.304			0.225
Vegetable oils only	858 (79.08%)	863 (78.24%)		297 (75.19%)	322 (77.03%)		561 (81.30%)	542 (79.01%)	
Mainly Vegetable oils	207 (19.08%)	222 (20.13%)		84 (21.27%)	89 (21.29%)		123 (17.83%)	133 (19.39%)	
50% Vegetable oils	17 (1.57%)	16 (1.45%)		12 (3.04%)	5 (1.20%)		5 (0.72%)	11 (1.60%)	
Mainly animal oils	3 (0.28%)	2 (0.18%)		2 (0.51%)	2 (0.48%)		1 (0.14%)	0 (0.00%)	
Consumption of Bean product, *n* (%)			0.991			0.626			0.815
< 1/week	546 (50.32%)	555 (50.18%)		145 (36.62%)	170 (40.48%)		401 (58.20%)	385 (56.04%)	
1–2/ week	349 (32.17%)	353 (31.92%)		154 (38.89%)	146 (34.76%)		195 (28.30%)	208 (30.28%)	
3–5/week	142 (13.09%)	146 (13.20%)		74 (18.69%)	80 (19.05%)		68 (9.87%)	66 (9.61%)	
Almost everyday	48 (4.42%)	52 (4.70%)		23 (5.81%)	24 (5.71%)		25 (3.63%)	28 (4.08%)	
Consumption of *Meat, n* (%)			0.598			0.829			0.578
< 1/week	505 (46.67%)	490 (44.30%)		139 (35.28%)	139 (33.10%)		366 (53.20%)	352 (51.24%)	
1–2/ week	372 (34.38%)	410 (37.07%)		145 (36.80%)	167 (39.76%)		227 (32.99%)	243 (35.37%)	
3–5/week	131 (12.11%)	135 (12.21%)		63 (15.99%)	63 (15.00%)		68 (9.88%)	72 (10.48%)	
Almost everyday	74 (6.84%)	71 (6.42%)		47 (11.93%)	51 (12.14%)		27 (3.92%)	20 (2.91%)	
Consumption of Vegetables and Fruits (per week), *n*(%)			0.248			0.137			0.268
< 0.5 kg	7 (0.65%)	15 (1.36%)		3 (0.76%)	7 (1.67%)		4 (0.58%)	8 (1.17%)	
0.5–1.5 kg	197 (18.17%)	197 (17.83%)		81 (20.45%)	67 (15.95%)		116 (16.86%)	131 (19.10%)	
> 1.5 kg	880 (81.18%)	893 (80.81%)		312 (78.79%)	346 (82.38%)		568 (82.56%)	547 (79.74%)	
Vitamin supplementation, *n* (%)			0.92			0.429			0.154
Never	1032 (97.54%)	1059 (97.60%)		369 (95.84%)	405 (97.83%)		663 (98.51%)	655 (97.47%)	
1–2/ week	5 (0.47%)	7 (0.65%)		5 (1.30%)	2 (0.48%)		0 (0.00%)	5 (0.74%)	
3–5/ week	7 (0.66%)	7 (0.65%)		4 (1.04%)	3 (0.72%)		3 (0.45%)	4 (0.60%)	
Almost everyday	14 (1.32%)	12 (1.11%)		7 (1.82%)	4 (0.97%)		7 (1.04%)	8 (1.19%)	
Education, *n* (%)			0.257			0.98			0.059
Illiterate	653 (60.35%)	676 (61.40%)		125 (31.65%)	133 (31.67%)		528 (76.86%)	543 (79.62%)	
Elementary or junior high school	235 (21.72%)	210 (19.07%)		118 (29.87%)	123 (29.29%)		117 (17.03%)	87 (12.76%)	
Senior high school or above	194 (17.93%)	215 (19.53%)		152 (38.48%)	164 (39.05%)		42 (6.11%)	52 (7.62%)	
Smoking, *n* (%)	327 (30.14%)	335 (30.29%)	0.987	290 (73.23%)	296 (70.48%)	0.426	37 (5.37%)	39 (5.68%)	0.896
Alcohol consumption, *n* (%)	319 (29.40%)	343 (31.04%)	0.438	272 (68.86%)	292 (69.52%)	0.897	47 (6.81%)	51 (7.43%)	0.731

*Data are presented as mean ± standard deviation or number (%). P-values were calculated using independent t-test for the continuous variables and chi-square test for categorical variables. tHcy, total homocysteine; BMI, body mass index; WHR, Waist-to-Hip Ratio; SBP, systolic blood pressure; DBP, diastolic blood pressure; MAP, mean arterial pressure; HDL-C, high-density lipoprotein cholesterol; PWV, pulse wave velocity; ALP, alkaline phosphatase; eGFR, estimated glomerular filtration rate; BUN, blood urea nitrogen*.

The tHcy levels were 5.51–114.37 μmol/L among all participants. Subjects were stratified into three groups according to tHcy levels: < 10, 10–15, and >15 μmol/L. General and biochemical characteristics of the study participants by tHcy level are displayed in Table 3. Comparing the different subgroups, by each sex and for the total participants, age (*P* < 0.001), uric acid (*P* < 0.001), creatinine (*P* < 0.001), and BUN (*P* < 0.001) increased from the lowest to the highest tHcy group, whereas estimated glomerular filtration rate (*P* < 0.001) decreased. Among the total participants, there were significant differences in smoking (*P* < 0.001) and alcohol consumption (*P* < 0.001) that increased from the lowest to the highest tHcy groups.

**Table 3:** Baseline characteristics by tHcy categories.

	**Total**			**Male**					**Female**		
**Characteristics**	**tHcy < 10 (umol/L)**	**10 = < tHcy < 15 (umol/L)**	**tHcy > 15 (umol/L)**	***P*-value**	**tHcy < 10 (umol/L)**	**10 = < tHcy < 15 (umol/L)**	**tHcy > 15 (umol/L)**	***P*-value**	**tHcy < 10 (umol/L)**	**10 = < tHcy < 15 (umol/L)**	**tHcy > 15 (umol/L)**	***P*-value**
	***n* = 540**	***n* = 1,159**	***n* = 500**		**109**	**441**	**267**		**431**	**718**	**233**	
Age, years	60.95 ± 6.53	64.27 ± 6.85	66.93 ± 7.41	< 0.001	61.05 ± 6.64	63.97 ± 7.25	66.94 ± 7.45	< 0.001	60.92 ± 6.50	64.45 ± 6.60	66.92 ± 7.38	< 0.001
BMI, kg/m^2^	26.33 ± 3.84	26.27 ± 3.88	25.73 ± 3.81	0.017	25.68 ± 4.02	25.46 ± 3.68	24.97 ± 3.76	0.134	26.49 ± 3.78	26.76 ± 3.93	26.60 ± 3.69	0.516
WHR	0.93 ± 0.06	0.94 ± 0.06	0.94 ± 0.07	< 0.001	0.94 ± 0.06	0.94 ± 0.06	0.94 ± 0.07	0.963	0.92 ± 0.06	0.94 ± 0.06	0.94 ± 0.07	< 0.001
SBP, mmHg	136.22 ± 16.36	137.00 ± 17.83	136.94 ± 18.12	0.680	134.52 ± 15.38	134.59 ± 18.41	134.38 ± 17.28	0.989	136.65 ± 16.60	138.49 ± 17.31	139.88 ± 18.65	0.055
DBP, mmHg	81.51 ± 10.69	81.13 ± 11.31	80.11 ± 10.98	0.107	83.82 ± 11.18	81.62 ± 11.88	80.28 ± 11.48	0.027	80.92 ± 10.49	80.82 ± 10.95	79.92 ± 10.38	0.472
MAP, mmHg	99.75 ± 10.82	99.75 ± 11.63	99.06 ± 11.46	0.490	100.72 ± 10.56	99.28 ± 12.24	98.32 ± 11.56	0.191	99.50 ± 10.89	100.04 ± 11.23	99.91 ± 11.31	0.724
Uric Acid, umol/L	280.84 ± 72.36	321.25 ± 84.62	391.29 ± 107.39	< 0.001	310.03 ± 80.18	346.91 ± 87.15	415.70 ± 106.17	< 0.001	273.46 ± 68.40	305.46 ± 79.05	363.33 ± 102.02	< 0.001
Creatinine, μmol/L	54.42 ± 12.40	63.93 ± 15.23	81.51 ± 29.07	< 0.001	65.98 ± 12.05	73.30 ± 13.35	90.08 ± 32.68	< 0.001	51.50 ± 10.68	58.16 ± 13.33	71.69 ± 20.31	< 0.001
Triglycerides, mmol/L	2.33 ± 1.76	2.34 ± 1.98	2.38 ± 2.08	0.907	2.11 ± 2.01	2.05 ± 1.88	2.19 ± 2.39	0.682	2.39 ± 1.69	2.52 ± 2.02	2.59 ± 1.62	0.327
HDL-C, mmol/L	1.25 ± 0.28	1.23 ± 0.30	1.32 ± 0.42	< 0.001	1.24 ± 0.32	1.23 ± 0.33	1.28 ± 0.42	0.181	1.25 ± 0.27	1.23 ± 0.28	1.36 ± 0.42	< 0.001
Glucose, mmol/L	9.60 ± 3.60	9.35 ± 3.09	8.96 ± 3.06	0.006	9.83 ± 3.93	9.37 ± 3.19	8.90 ± 2.67	0.023	9.55 ± 3.51	9.33 ± 3.03	9.04 ± 3.47	0.156
ALP, U/L	96.39 ± 29.41	98.39 ± 30.96	98.60 ± 33.21	0.404	85.32 ± 26.22	86.68 ± 25.32	90.56 ± 28.68	0.102	99.18 ± 29.54	105.52 ± 31.91	107.78 ± 35.62	< 0.001
eGFR, ml/min/1.73m^2^	99.59 ± 10.69	91.56 ± 12.94	78.27 ± 18.86	< 0.001	99.45 ± 10.67	92.11 ± 12.18	79.16 ± 20.07	< 0.001	99.62 ± 10.71	91.22 ± 13.38	77.26 ± 17.35	< 0.001
BUN, mmol/L	5.57 ± 1.36	6.19 ± 1.65	7.25 ± 2.57	< 0.001	5.98 ± 1.57	6.38 ± 1.66	7.28 ± 2.68	< 0.001	5.47 ± 1.28	6.07 ± 1.63	7.22 ± 2.44	< 0.001
PWV, mm/s	1696.42 ± 349.16	1785.98 ± 402.35	1830.20 ± 448.21	< 0.001	1674.11 ± 383.30	1734.71 ± 380.27	1770.49 ± 476.02	0.151	1701.97 ± 340.45	1817.14 ± 412.37	1896.76 ± 405.86	< 0.001
Retinal arteriosclerosis, *n* (%)	393 (72.78%)	898 (77.48%)	407 (81.40%)	0.004	81 (74.31%)	353 (80.05%)	220 (82.40%)	0.205	312 (72.39%)	545 (75.91%)	187 (80.26%)	0.075
Cooking oil, *n* (%)				0.036				0.336				0.154
Vegetable oils only	441 (81.82%)	895 (77.62%)	386 (77.67%)		87 (79.82%)	334 (76.26%)	198 (74.44%)		354 (82.33%)	561 (78.46%)	188 (81.39%)	
Mainly vegetable oils	95 (17.63%)	228 (19.77%)	106 (21.33%)		22 (20.18%)	87 (19.86%)	64 (24.06%)		73 (16.98%)	141 (19.72%)	42 (18.18%)	
50% Vegetable oils	2 (0.37%)	27 (2.34%)	4 (0.80%)		0 (0.00%)	14 (3.20%)	3 (1.13%)		2 (0.47%)	13 (1.82%)	1 (0.43%)	
Mainly animal oils	1 (0.19%)	3 (0.26%)	1 (0.20%)		0 (0.00%)	3 (0.68%)	1 (0.38%)		1 (0.23%)	0 (0.00%)	0 (0.00%)	
Consumption of Bean product, *n* (%)				0.871				0.037				0.521
< 1/ week	270 (50.09%)	570 (49.35%)	261 (52.41%)		34 (31.19%)	158 (35.83%)	123 (46.24%)		236 (54.88%)	412 (57.70%)	138 (59.48%)	
1–2/ week	170 (31.54%)	377 (32.64%)	156 (31.33%)		45 (41.28%)	164 (37.19%)	91 (34.21%)		125 (29.07%)	213 (29.83%)	65 (28.02%)	
3–5/ week	74 (13.73%)	151 (13.07%)	63 (12.65%)		21 (19.27%)	90 (20.41%)	43 (16.17%)		53 (12.33%)	61 (8.54%)	20 (8.62%)	
Almost everyday	25 (4.64%)	57 (4.94%)	18 (3.61%)		9 (8.26%)	29 (6.58%)	9 (3.38%)		16 (3.72%)	28 (3.92%)	9 (3.88%)	
Consumption of Meat, *n* (%)				0.507				< 0.001				0.057
< 1/ week	238 (44.24%)	515 (44.67%)	243 (48.80%)		21 (19.44%)	149 (33.86%)	108 (40.60%)		217 (50.47%)	366 (51.33%)	135 (58.19%)	
1–2/ week	198 (36.80%)	411 (35.65%)	173 (34.74%)		48 (44.44%)	165 (37.50%)	99 (37.22%)		150 (34.88%)	246 (34.50%)	74 (31.90%)	
3–5/ week	65 (12.08%)	143 (12.40%)	58 (11.65%)		25 (23.15%)	61 (13.86%)	40 (15.04%)		40 (9.30%)	82 (11.50%)	18 (7.76%)	
Almost everyday	37 (6.88%)	84 (7.29%)	24 (4.82%)		14 (12.96%)	65 (14.77%)	19 (7.14%)		23 (5.35%)	19 (2.66%)	5 (2.16%)	
Consumption of vegetables and fruits (per week), *n* (%)				0.261				0.412				0.426
< 0.5 kg	3 (0.56%)	10 (0.87%)	9 (1.81%)		1 (0.92%)	3 (0.68%)	6 (2.26%)		2 (0.47%)	7 (0.98%)	3 (1.29%)	
0.5–1.5 kg	91 (16.95%)	211 (18.27%)	93 (18.67%)		21 (19.27%)	83 (18.82%)	44 (16.54%)		70 (16.36%)	128 (17.93%)	49 (21.12%)	
>1.5 kg	443 (82.50%)	934 (80.87%)	396 (79.52%)		87 (79.82%)	355 (80.50%)	216 (81.20%)		356 (83.18%)	579 (81.09%)	180 (77.59%)	
Vitamin supplementation, *n* (%)				0.375				0.369				0.147
Never	518 (98.48%)	1098 (97.25%)	476 (97.34%)		105 (97.22%)	415 (96.29%)	254 (97.69%)		413 (98.80%)	683 (97.85%)	222 (96.94%)	
1–2/ week	0 (0.00%)	10 (0.89%)	2 (0.41%)		0 (0.00%)	6 (1.39%)	1 (0.38%)		0 (0.00%)	4 (0.57%)	1 (0.44%)	
3–5/ week	2 (0.38%)	8 (0.71%)	4 (0.82%)		2 (1.85%)	2 (0.46%)	3 (1.15%)		0 (0.00%)	6 (0.86%)	1 (0.44%)	
Almost everyday	6 (1.14%)	13 (1.15%)	7 (1.43%)		1 (0.93%)	8 (1.86%)	2 (0.77%)		5 (1.20%)	5 (0.72%)	5 (2.18%)	
Education, *n* (%)				0.078				< 0.001				0.197
Illiterate	351 (65.49%)	681 (59.17%)	297 (59.76%)		30 (27.52%)	121 (27.50%)	107 (40.23%)		321 (75.18%)	560 (78.76%)	190 (82.25%)	
Elementary or junior high school	99 (18.47%)	235 (20.42%)	111 (22.33%)		30 (27.52%)	127 (28.86%)	84 (31.58%)		69 (16.16%)	108 (15.19%)	27 (11.69%)	
Senior high school or above	86 (16.04%)	235 (20.42%)	89 (17.91%)		49 (44.95%)	192 (43.64%)	75 (28.20%)		37 (8.67%)	43 (6.05%)	14 (6.06%)	
Smoking, *n* (%)	92 (17.07%)	362 (31.34%)	208 (41.77%)	< 0.001	78 (71.56%)	319 (72.34%)	189 (71.05%)	0.933	14 (3.26%)	43 (6.02%)	19 (8.19%)	0.021
Alcohol consumption, *n* (%)	103 (19.11%)	359 (31.11%)	200 (40.16%)	< 0.001	81 (74.31%)	303 (68.86%)	180 (67.67%)	0.438	22 (5.12%)	56 (7.84%)	20 (8.62%)	0.138

*Data are presented as mean ± standard deviation or number (%). P-values were calculated using independent t-test for the continuous variables and chi-square test for categorical variables. tHcy, total homocysteine; BMI, body mass index; WHR, Waist-to-Hip Ratio; SBP, systolic blood pressure; DBP, diastolic blood pressure; MAP, mean arterial pressure; HDL-C, high-density lipoprotein cholesterol; PWV, pulse wave velocity; ALP, alkaline phosphatase; eGFR, estimated glomerular filtration rate; BUN, blood urea nitrogen*.

To further explore the possible correlation between folic acid supplementation and RA, we conducted a stratified analysis and a logistic regression analysis. As a binary variable, compared with the enalapril group in the tHcy > 15 μmol/L group of females, the OR for the enalapril–folic acid group was 0.42 (95% CI, 0.20–0.85; *P* = 0.0191). Those associations remained unchanged after further adjustment. After adjustment, the OR for the enalapril–folic acid group was 0.28 (95% CI, 0.11–0.67; *P* = 0.0061), indicating that the enalapril–folic acid group had a 72% lower odds of RA in the tHcy > 15 μmol/L group of females (Table 4).

**Table 4:** Associations of folic acid intake with retinal arteriosclerosis.

**tHcy**	<**10 (umol/L)**		**10–15 (umol/L)**		>**15 (umol/L)**		**Overall**	
	**OR (95% CI)**	***P*-value**	**OR (95% CI)**	***P*-value**	**OR (95% CI)**	***P*-value**	**OR (95% CI)**	***P*-value**
**TOTAL**								
Unadjusted	1.06 (0.72, 1.56)	0.7655	0.92 (0.70, 1.21)	0.5514	0.72 (0.45, 1.15)	0.1722	0.95 (0.78, 1.16)	0.6197
Model 1	1.07 (0.72, 1.59)	0.7341	0.92 (0.69, 1.22)	0.5591	0.67 (0.41, 1.08)	0.1068	0.93 (0.76, 1.14)	0.4899
Model 2	1.07 (0.72, 1.59)	0.7404	0.93 (0.70, 1.23)	0.6097	0.68 (0.41, 1.08)	0.1085	0.94 (0.76, 1.15)	0.5163
Model 3	1.08 (0.71, 1.66)	0.7270	0.96 (0.71, 1.29)	0.7820	0.63 (0.38, 1.03)	0.0674	0.94 (0.76, 1.17)	0.5917
Model 4	0.97 (0.62, 1.52)	0.9065	0.94 (0.69, 1.27)	0.6732	0.65 (0.39, 1.07)	0.0966	0.92 (0.74, 1.15)	0.4679
Model 5	0.97 (0.61, 1.50)	0.8522	0.93 (0.68, 1.26)	0.6365	0.62 (0.37, 1.03)	0.0699	0.91 (0.73, 1.13)	0.4031
Model 6	0.96 (0.61, 1.50)	0.3605	0.92 (0.67, 1.25)	0.5926	0.64 (0.37, 1.04)	0.0766	0.90 (0.73, 1.13)	0.3700
**MALE**								
Unadjusted	1.53 (0.62, 3.78)	0.3605	1.09 (0.68, 1.74)	0.7210	1.14 (0.61, 2.16)	0.6758	1.20 (0.85, 1.70)	0.2941
Model 1	1.49 (0.57, 3.88)	0.4182	1.06 (0.66, 1.72)	0.8071	1.14 (0.59, 2.20)	0.6991	1.16 (0.82, 1.65)	0.3971
Model 2	1.42 (0.53, 3.81)	0.4804	1.07 (0.66, 1.73)	0.7927	1.13 (0.58, 2.19)	0.7132	1.16 (0.82, 1.64)	0.4136
Model 3	1.33 (0.47, 3.79)	0.5902	1.20 (0.72, 2.00)	0.4802	1.10 (0.54, 2.21)	0.7923	1.20 (0.83, 1.73)	0.3397
Model 4	1.02 (0.31, 3.34)	0.9703	1.24 (0.72, 2.14)	0.4364	1.23 (0.59, 2.56)	0.5746	1.27 (0.87, 1.86)	0.2205
Model 5	1.19 (0.32, 4.41)	0.7948	1.25 (0.72, 2.20)	0.4236	1.17 (0.55, 2.47)	0.6876	1.22 (0.83, 1.80)	0.3088
Model 6	1.04 (0.26, 4.09)	0.9573	1.28 (0.73, 2.26)	0.3891	1.20 (0.56, 2.57)	0.6386	1.23 (0.84, 1.82)	0.2938
**FEMALE**								
Unadjusted	0.98 (0.64, 1.50)	0.9178	0.84 (0.60, 1.19)	0.3276	0.42 (0.20, 0.85)	0.0191	0.84 (0.66, 1.07)	0.1595
Model 1	0.99 (0.64, 1.54)	0.9711	0.84 (0.59, 1.20)	0.3390	0.36 (0.16, 0.74)	0.0076	0.83 (0.64, 1.06)	0.1337
Model 2	1.00 (0.64, 1.56)	0.9962	0.85 (0.60, 1.22)	0.3839	0.35 (0.16, 0.74)	0.0078	0.83 (0.65, 1.07)	0.1494
Model 3	1.06 (0.66, 1.73)	0.7996	0.87 (0.60, 1.26)	0.4528	0.32 (0.14, 0.69)	0.0054	0.83 (0.64, 1.08)	0.1668
Model 4	0.92 (0.55, 1.53)	0.7414	0.82 (0.56, 1.21)	0.3112	0.29 (0.11, 0.66)	0.0051	0.77 (0.59, 1.01)	0.0582
Model 5	0.92 (0.55, 1.54)	0.7553	0.80 (0.54, 1.18)	0.2604	0.28 (0.11, 0.67)	0.0059	0.77 (0.59, 1.01)	0.0596
Model 6	0.92 (0.55, 1.54)	0.7479	0.79 (0.53, 1.16)	0.2293	0.28 (0.11, 0.67)	0.0061	0.77 (0.58, 1.00)	0.0544

*Model 1: adjusted for age, SBP, DBP, BMI, WHR, education. Model 2: adjusted for age, SBP, DBP, BMI, WHR, education, smoking, alcohol consumption. Model 3: adjusted for age, SBP, DBP, BMI, WHR, education, smoking, alcohol consumption, PWV, MAP. Model 4: adjusted for age, SBP, DBP, BMI, WHR, education, smoking, alcohol consumption, PWV, MAP, cooking oil, consumption of bean product, consumption of meat, consumption of vegetables and fruits, vitamin supplementation. Model 5: adjusted for age, SBP, DBP, BMI, WHR, education, smoking, alcohol consumption, PWV, MAP, cooking oil, consumption of bean product, consumption of meat, consumption of vegetables and fruits, vitamin supplementation, Uric acid, Creatinine, eGFR, ALP, BUN. Model 6: adjusted for age, SBP, DBP, BMI, WHR, education, smoking, alcohol consumption, PWV, MAP, cooking oil, consumption of bean product, consumption of meat, consumption of vegetables and fruits, vitamin supplementation, Uric acid, Creatinine, eGFR, ALP, BUN, Glucose; Triglycerides, HDL-C*.

*SBP, systolic blood pressure; DBP, diastolic blood pressure; BMI, body mass index; WHR, Waist-to-Hip Ratio; PWV, pulse wave velocity; MAP, mean arterial pressure; eGFR, estimated glomerular filtration rate; ALP, alkaline phosphatase; BUN, blood urea nitrogen; HDL-C, high-density lipoprotein cholesterol*.

To explore the potential interactions between folic acid supplementation and related factors on the risk of RA, further stratified analyses were conducted (Table 5). After the adjustment of variables, the result showed that among non-smokers with a tHcy > 15 μmol/L, the enalapril–folic acid group had a 57% lower risk of RA (OR, 0.43; 95% CI, 0.20–0.88; *P* = 0.0248) than the enalapril group. Among non-drinkers with a tHcy > 15 μmol/L, folic acid supplementation lowered the risk of RA by 53% (OR, 0.47; 95% CI, 0.23–0.94; *P* = 0.0375). For patients aged 55–65 years with a tHcy > 15 μmol/L, folic acid supplementation was associated with a 73% lower risk of RA (OR, 0.27; 95% CI, 0.08–0.79; *P* = 0.0225). For those with a BMI of 24.9–29.9 and a tHcy > 15 μmol/L, folic acid supplementation was associated with a 66% lower risk of RA (OR, 0.34; 95% CI, 0.13–0.87; *P* = 0.0291).

**Table 5:** Logistic regression analysis of folic acid supplementation in relation to RA in different subgroups.

	***n***	**tHcy**<**10 (umol/L)**		**10** = <**tHcy**<**15 (umol/L)**		**tHcy** >**15 (umol/L)**		**Overall**	
		**OR (95% CI)**	***P*-value**	**OR (95% CI)**	***P*-value**	**OR (95% CI)**	***P*-value**	**OR (95% CI)**	***P*-value**
**AGE, y**									
< 55	249	0.18 (0.02, 1.10)	0.0949	2.77 (0.73, 11.5)	0.1415	NA	NA	1.10 (0.54, 2.24)	0.7938
55-65	976	1.27 (0.87, 2.36)	0.4497	0.94 (0.57, 1.54)	0.8073	0.27 (0.08, 0.79)	0.0225	0.92 (0.66, 1.28)	0.6069
>65	974	0.86 (0.31, 2.41)	0.7784	0.71 (0.44, 1.12)	0.1407	0.91 (0.44, 1.84)	0.7926	0.80 (0.57, 1.12)	0.1846
**BMI, kg/m**^2^									
< 24.9	836	0.85 (0.37, 1.99)	0.7146	1.18 (0.68, 2.17)	0.5211	0.86 (0.33, 2.14)	0.7769	1.02 (0.70, 1.50)	0.9109
24.9-29.9	1,019	1.25 (0.63, 2.51)	0.5284	0.88 (0.56, 1.39)	0.5808	0.34 (0.13, 0.87)	0.0291	0.90 (0.65, 1.24)	0.5128
>29.9	344	0.64 (0.12, 3.05)	0.5804	0.49 (0.21, 1.15)	0.1056	NA	NA	0.66 (0.37, 1.16)	0.1468
**SMOKING**									
No	1,530	0.91 (0.55, 1.52)	0.7158	0.76 (0.52, 1.10)	0.1404	0.43 (0.20, 0.88)	0.0248	0.75 (0.58, 0.98)	0.0304
Yes	662	1.30 (0.37, 4.76)	0.6799	1.60 (0.88, 2.95)	0.1227	1.68 (0.65, 4.43)	0.2859	1.50 (0.99, 2.29)	0.0554
**ALCOHOL DRINKING**									
No	1,529	0.85 (0.51, 1.41)	0.5315	0.80 (0.55, 1.16)	0.2382	0.47 (0.23, 0.94)	0.0375	0.76 (0.59, 0.99)	0.0430
Yes	662	3.51 (0.89, 16.89)	0.0896	1.22 (0.67, 2.23)	0.5088	0.99 (0.39, 2.44)	0.9760	1.33 (0.88, 2.03)	0.1797

*Adjusted for sex, SBP, DBP, WHR, education, PWV, MAP, cooking oil, consumption of bean product, consumption of meat, consumption of vegetables and fruits, vitamin supplementation, Uric acid, Creatinine, eGFR, ALP, BUN, Glucose; Triglycerides, HDL-C*.

*SBP, systolic blood pressure; DBP, diastolic blood pressure, BMI, body mass index; WHR, Waist-to-Hip Ratio; PWV, pulse wave velocity; MAP, mean arterial pressure; eGFR, estimated glomerular filtration rate; ALP, alkaline phosphatase; BUN, blood urea nitrogen; HDL-C, high-density lipoprotein cholesterol*.

## Discussion

This was the first community-based epidemiologic study of RA in rural China. Our results provide valuable information about the epidemiology of RA in a population with hypertension complicated by diabetes mellitus. This cross-sectional study of 2,199 participants with hypertension complicated by diabetes mellitus showed a positive correlation between folic acid supplementation and the risk of RA in females with hyperhomocysteinemia.

Increasing studies (Temple et al., 2000; Antoniades et al., 2009) have shown that hyperhomocysteinemia, characterized by an abnormally high level of tHcy (>15 μmol/L) (Sacco et al., 2006; Guo et al., 2009), is closely related to atherosclerosis. Hyperhomocysteinemia causes vascular dysfunction in two different ways (Sen et al., 2010). First, Hcy in the blood increases BP. Second, it activates metalloproteinase, which triggers the formation of collagen, causing an imbalance in the ratio of elasticity to collagen and reducing blood vessel elasticity. The metabolite of Hcy is hydrogen sulfide (H2S), a strong antioxidant and endothelium-derived relaxation factor. However, in the case of hyperhomocysteinemia, as the Hcy level increases, H2S production is decreased as cystathionine-γ-synthase is inhibited, causing hypertension and diabetes. Several studies have proven that hypertension and hyperhomocysteinemia have a synergistic effect for causing cardiovascular and cerebrovascular events. Liu's research (Liu et al., 2018) proved that comorbid prehypertension and hyperhomocysteinemia was an independent risk factor of subclinical atherosclerosis in asymptomatic Chinese adults, whereas isolated prehypertension or hyperhomocysteinemia was not. Graham's study (Graham et al., 1997) showed that hypertension complicated by hyperhomocysteinemia increased the risk of vessel diseases by 11 times (relative risk [RR] = 11.3).

Folic acid supplementation is currently the safest and most effective way to lower Hcy levels (Pezzini et al., 2007). Tetrahydrofolate, which can be formed from supplemental folic acid under the impact of dihydrofolate reductase and vitamin B12, carries one-carbon units to cause methylation in Hcy to form methionine, thus lowering Hcy levels in the blood (Stipanuk, 2004). In a meta-analysis (Wang et al., 2007) of clinical trials evaluating the efficacy of folate supplementation for stroke prevention, folate was associated with an 18% reduction (RR = 0.82; 95% CI, 0.68–1.00) in primary stroke risk. In the CSPPT study (Huo et al., 2015), the risk of stroke was reduced by 25% (RR = 0.75; 95% CI, 0.62–0.90). The reduction of risk became even more significant after the participants took folic acid for >36 months (RR = 0.71; 95% CI, 0.57–0.87) and the Hcy level decreased by 20% (RR = 0.77; 95% CI, 0.63–0.94). The Heart Outcomes Prevention Evaluation-2 (HOPE-2) trial (Saposnik et al., 2009) showed that folic acid supplementation significantly reduced the risk of overall stroke (hazard ratio [HR], 0.75; 95% CI, 0.59–0.97) and non-fatal stroke (HR, 0.72; 95% CI, 0.54–0.95). Also, people in the areas where the population did not consume supplemental folic acid (HR, 0.67; 95% CI, 0.46–0.97) and had higher Hcy levels (>13.8 μmol/L; HR, 0.57; 95% CI, 0.33–0.97) as well as those who had histories of stroke or transient ischemic attacks benefit more from folic acid supplementation.

The increasing activity of angiotensin converting enzymes (ACE) also contribute to hyperhomocysteinemia leads to hypertension (Poduri et al., 2008). Therefore, ACE activity should be inhibited, and antihypertensives with ACEI should be used while lowering Hcy in medical treatment (Dierkes et al., 2007). In the HOPE-2 study (Liakishev, 2006), 5,522 subjects ≥55 years of age with cardiovascular or cerebrovascular diseases or diabetes were given a vitamin B compound (2.5 mg of folic acid, 50 mg of vitamin B6, and 1 mg of vitamin B12) or placebo; 65% of the subjects were taking ACEI at the same time. During 5-year follow-up, the differential of Hcy of the two groups was 3.2 μmol/L, and a significant 25% decrease in stroke risk was seen in the group that took vitamin B with folic acid at the endpoint (HR, 0.75; 95% CI, 0.59–0.97). In the WAFACS study (Albert et al., 2008), the result showed that ACEI and folic acid have a synergistic effect in preventing cardiovascular or cerebrovascular events. The patients benefited by 19% (*P* = 0.03%). In conclusion, patients benefited more when folic acid is supplemented in addition to antihypertensives with ACEI.

A single-blind randomized controlled trial and a meta-analysis (Wald et al., 2001; Homocysteine Lowering Trialists' Collaboration, 2005) indicated that 0.8 mg of folic acid per day can have the best effect at lowering Hcy (the suggested maximum tolerance dose of folic acid by the United States Food and Nutrition Board is 1 mg per day). In our study, the enalapril–folic acid group took 0.8 mg of folic acid and 10 mg of enalapril per day. The CSPPT study (Huo et al., 2015) found that the enalapril–folic acid group had a significant risk reduction in first stroke compared with the enalapril-alone group (2.7% of participants in the enalapril–folic acid group vs. 3.4% in the enalapril-alone group; HR, 0.79; 95% CI, 0.68–0.93). Our study also proved that in female patients with hyperhomocysteinemia (Hcy > 15 μmol/L) and hypertension complicated by diabetes, folic acid supplementation lowered the risk of RA by 72% (OR, 0.28; 95% CI, 0.11–0.67; *P* = 0.0061).

Folates also contribute to enhanced endothelial function by increasing NO bioavailability within the vascular endothelium, thereby preventing or reversing the progression of cerebrovascular disease (Stanhewicz and Kenney, 2017).

We observed an interesting phenomena that folic acid supplementation may have a greater influence in female. Much experimental and epidemiological evidence reported that estrogen exert a vascular protective function and may have beneficial effects on endothelial function and atherosclerosis, raising the possibility of sex differences in arterial remodeling (Iorga et al., 2017). The difference we saw between male and females might because of their different lifestyles such as smoking and drinking.

Study showed that smoking affected the absorption of folic acid (Ortega et al., 1994). Smokers had lower levels of folic acid than non-smokers (Chen et al., 2015). Study showed drinking alcohol affected metabolism of Hcy, raising the level of Hcy (van der Gaag et al., 2000; Gonzalez-Ortiz et al., 2005). Gibson's research (Gibson et al., 2008) found that drinking alcohol lowered the levels of folid acid and vitamin B12 and raised the level of blood Hcy. In our study, 71.85% of the males and 5.52% of the females were smokers, 69.12% of the males and 7.11% of the females drank alcohol. This may be a possible explanation for the differences in associations of folic acid supplementaion and RA between males and females in our cohort.

The advantage of our study is that, although there have been many studies of the effect of folic acid consumption on reducing the risk or slowing the progress of atherosclerosis, hypertension, and stroke, no study has examined the correlation between RA and folic acid supplementation in patients with hypertension complicated by diabetes. Our study is the first on this subject. On the other hand, our study is a nested cross-sectional study from CSPPT, in which 20,702 essential hypertension patients were given enalapril and folic acid or enalapril-only double-blind treatment for 5 years to analyze the differences in the incidence of stroke and other vessel diseases at the endpoint.

Our study had limitations. First, although we considered many factors such as age, sex, BP, blood, and cholesterol levels and adjusted for them accordingly, we failed to consider factors such as MTHFR genotyping. Second, since folic acid supplementation is not common in China, although the significant effect of enalapril—folic acid is seen in rural Chinese populations in which microelement supplementation is insufficient, whether the same effect can be seen in urban areas where folic acid supplementation is more common has yet to be evaluated.

In conclusion, this cross-sectional study demonstrated that folic acid supplementation was significantly associated with RA and a high tHcy level in female. Folic acid supplementation lowered the risk of RA in females with hyperhomocysteinemia and hypertension complicated by diabetes. No significant association were seen in males.

## Author contributions

LY, YH, XX, BW, and RL: study design; JiL, FH: literature research; KS, XC, YZ, XG, and GT: data acquisition; XQ: data analysis; JuL, HS, LZ, YP, JZ, and YM: fundus photographs analysis; HL and WY: statistical analysis; YM: manuscript preparation.

### Conflict of interest statement

The authors declare that the research was conducted in the absence of any commercial or financial relationships that could be construed as a potential conflict of interest.
