# Supplementary material for: Association Between Folic Acid Supplementation and Retinal Atherosclerosis in Chinese Adults With Hypertension Complicated by Diabetes Mellitus
Source: Front Pharmacol. 2018 Oct 30;9:1159. doi: 10.3389/fphar.2018.01159 (PMC6218923; doi:10.3389/fphar.2018.01159)
Supplement: Supplementary file 1 [file Table_1.DOC]

Supplement Table 1. Baseline Characteristics of the Study Participants

| Characteristics | Male | Female | *P* value |
| --- | --- | --- | --- |
|  | n=817 | n=1382 |  |
| Age, years | 64.55 ± 7.47 | 63.78 ± 7.03 | 0.011 |
| BMI, kg/m^2^ | 25.33 ± 3.75 | 26.65 ± 3.85 | <0.001 |
| WHR | 0.94 ± 0.06 | 0.93 ± 0.06 | 0.001 |
| SBP, mmHg | 134.50 ± 17.64 | 138.16 ± 17.34 | <0.001 |
| DBP, mmHg | 81.48 ± 11.69 | 80.68 ± 10.71 | 0.159 |
| MAP, mmHg | 99.16 ± 11.81 | 99.84 ± 11.13 | 0.176 |
| tHcy, umol/L | 15.31 ± 9.11 | 12.14 ± 4.66 | <0.001 |
| Uric acid, umol/L | 364.47 ± 100.21 | 305.24 ± 85.65 | <0.001 |
| Creatinine, μmol/L | 77.81 ± 23.29 | 58.37 ± 15.55 | <0.001 |
| Triglycerides, mmol/L | 2.11 ± 2.07 | 2.49 ± 1.86 | <0.001 |
| HDL-C, mmol/L | 1.25 ± 0.36 | 1.26 ± 0.31 | 0.053 |
| Glucose, mmol/L | 9.28 ± 3.16 | 9.35 ± 3.27 | 0.999 |
| ALP, U/L | 87.77 ± 26.63 | 103.93 ± 32.01 | <0.001 |
| eGFR, ml/min/1.73m^2^ | 88.86 ± 16.67 | 91.48 ± 15.30 | <0.001 |
| BUN, mmol/L | 6.62 ± 2.09 | 6.08 ± 1.79 | <0.001 |
| PWV, mm/s | 1738.46 ± 415.18 | 1795.79 ± 395.99 | 0.005 |
| Retinal arteriosclerosis, n( %) | 654 (79.95%) | 1046 (75.58%) | 0.018 |
| Cooking oil, n( %) |  |  | 0.026 |
| [Vegetable oils only](http://dict.cnki.net/dict_result.aspx?searchword=植物油&tjType=sentence&style=&t=vegetable+oil) | 619 (76.14%) | 1104 (80.12%) |  |
| [Mainly Vegetable oils](http://dict.cnki.net/dict_result.aspx?searchword=植物油&tjType=sentence&style=&t=vegetable+oil) | 173 (21.28%) | 256 (18.58%) |  |
| [50% Vegetable oils](http://dict.cnki.net/dict_result.aspx?searchword=植物油&tjType=sentence&style=&t=vegetable+oil) | 17 ( 2.09%) | 17 ( 1.23%) |  |
| Mainly animal oils | 4 ( 0.49%) | 1 ( 0.07%) |  |
| Consumption of Bean product, n( %) |  |  | <0.001 |
| <1/ week | 316 (38.68%) | 788 (57.18%) |  |
| 1-2/ week | 300 (36.72%) | 403 (29.25%) |  |
| 3-5/ week | 154 (18.85%) | 134 ( 9.72%) |  |
| Almost everyday | 47 ( 5.75%) | 53 ( 3.85%) |  |
| Consumption of Meat, n( %) |  |  | <0.001 |
| <1/ week | 279 (34.23%) | 720 (52.29%) |  |
| 1-2/ week | 312 (38.28%) | 470 (34.13%) |  |
| 3-5/ week | 126 (15.46%) | 140 (10.17%) |  |
| Almost everyday | 98 (12.02%) | 47 ( 3.41%) |  |
| Consumption of Vegetables and Fruits( per week), n( %) |  |  | 0.711 |
| < 0.5kg | 10 ( 1.22%) | 12 ( 0.87%) |  |
| 0.5- 1.5kg | 149 (18.24%) | 247 (17.95%) |  |
| > 1.5kg | 658 (80.54%) | 1117 (81.18%) |  |
| Vitamin supplementation, n( %) |  |  | 0.308 |
| never | 775 (96.88%) | 1320 (98.00%) |  |
| 1-2/ week | 7 ( 0.88%) | 5 ( 0.37%) |  |
| 3-5/ week | 7 ( 0.88%) | 7 ( 0.52%) |  |
| Almost everyday | 11 ( 1.38%) | 15 ( 1.11%) |  |
| Education, n( %) |  |  | <0.001 |
| Illiterate | 258 (31.62%) | 1072 (78.19%) |  |
| Elementary or junior high school | 242 (29.66%) | 205 (14.95%) |  |
| Senior high school or above | 316 (38.73%) | 94 ( 6.86%) |  |
| Smoking, n( %) | 587 (71.85%) | 76 ( 5.52%) | <0.001 |
| Alcohol consumption, n( %) | 564 (69.12%) | 98 ( 7.11%) | <0.001 |

Abbreviations: tHcy total homocysteine, BMI, body mass index, WHR Waist-to-Hip Ratio, SBP systolic blood pressure, DBP diastolic blood pressure, MAP mean arterial pressure, HDL-C high-density lipoprotein cholesterol, PWV pulse wave velocity, ALP alkaline phosphatase, eGFR, estimated glomerular filtration rate, BUN blood urea nitrogen
